# Warfarin use in hemodialysis patients with atrial fibrillation: decisions based on uncertainty

**DOI:** 10.1186/1471-2369-14-174

**Published:** 2013-08-13

**Authors:** Salima Juma, Benjamin KA Thomson, Charmaine E Lok, Catherine M Clase, Peter G Blake, Louise Moist

**Affiliations:** 1 Kidney Clinical Research Unit, Schulich School of Medicine and Dentistry, Western University, 800 Commissioners Rd E, London, Ontario N6A 5W9, Canada; 2 Division of Nephrology, Department of Medicine, London Health Sciences Center, London, Ontario, Canada; 3 Division of Nephrology, Department of Medicine, Toronto General Hospital, University of Toronto, Toronto, Ontario, Canada; 4 Department of Medicine, McMaster University, Hamilton, Ontario, Canada; 5 Department of Clinical Epidemiology and Statistics, McMaster University, Hamilton, Ontario, Canada

**Keywords:** Warfarin, Anticoagulation, Atrial fibrillation, Hemodialysis, Stroke, CKD, Bleeding

## Abstract

**Background:**

Warfarin prescribing patterns for hemodialysis patients with atrial fibrillation vary widely amongst nephrologists. This may be due to a paucity of guiding evidence, but also due to concerns of increased risks of warfarin use in this population. The literature lacks clarity on the balance of warfarin therapy between prevention of thrombotic strokes and the increased risks of bleeding in hemodialysis patients with atrial fibrillation.

**Methods:**

We performed a survey of Canadian Nephrologists, assessing warfarin prescribing practice, and measured the certainty in making these choices.

**Results:**

Respondents were consistently uncertain about warfarin use for atrial fibrillation. This uncertainty increased with a history of falls or starting hemodialysis, even when a high CHADS2 or CHA2DS2VASc score was present. The majority of respondents agreed that clinical equipoise existed about the use of oral anticoagulation in hemodialysis patients with atrial fibrillation (72.2%) and that the results of a randomized controlled trial would be relevant to their practice (98.2%).

**Conclusions:**

A randomized controlled trial of warfarin use in hemodialysis patients with atrial fibrillation would clarify the risks and benefits of warfarin use in this population.

## Background

Warfarin is indicated in the general population for the treatment of venous thromboembolism (VTE) and for prophylaxis of stroke in patients with atrial fibrillation. A recent non-randomized retrospective database analysis confirmed that in patients with non-valvular atrial fibrillation and chronic kidney disease, the use of warfarin or aspirin reduced stroke but increased risk of bleeding [1]. However, in patients on dialysis, warfarin is widely used for nonvalvular atrial fibrillation, though the ratio of risks to benefits remains unclear [2-6]. Warfarin has not been studied in randomized trials in this population, and trials of low-intensity warfarin for the maintenance of access patency showed no benefit [7,8].

The prevalence of atrial fibrillation in patients with end-stage kidney disease on hemodialysis is higher than that in the general population, ranging from 7 to 27% [9-12]. In the United States, the prevalence has tripled from 1992 to 2006. These patients have increased risks for adverse effects from warfarin: major bleeding including hemorrhagic stroke [2,13-15], acceleration of vascular calcification, calcific uremic arteriolopathy and warfarin-induced skin necrosis [16-18]. Patients with severe kidney disease were excluded in the generation and validation of stroke and bleeding risk calculator scores such as CHADS2 and CHA2DS2VASc [19-22], which further raises uncertainty about whether evidence from the general population can be generalized to people on dialysis.

Internationally, warfarin prescribing patterns for hemodialysis patients with atrial fibrillation vary widely, from 2% in Germany to as high as 37% in Canada [6]. Since Canadian nephrologists prescribe warfarin frequently in this situation, the objective of our study was to identify the degree of uncertainty and clinical equipoise in these decisions for treatment, and to assess support for a randomized trial.

## Methods

### National survey of nephrologists

We developed a survey consisting of six clinically-relevant cases, followed by four questions. The cases were designed to include different standardized CHADS2 and CHA2DS2VASc ischemic stroke risk scores, and variable bleeding risk, and were reviewed independently by three nephrologists for clinical validity. The additional questions explicitly evaluated respondents’ sense of clinical equipoise, their willingness to enter patients with atrial fibrillation into a randomized controlled trial, and their perceived utility of a randomized control trial of anticoagulation of hemodialysis patients with atrial fibrillation. Respondents were also asked 6 demographic questions, to assess representativeness.

We distributed the survey, using Survey Monkey, to a random sample of members of the Canadian Society of Nephrology. We chose to survey one-third, rather than the whole membership to reduce respondent burden and maximize the response rate. The identical survey was sent three separate times within 6 weeks, at 2 week intervals. Only one response per respondent was permitted.

Fisher’s exact test was used to establish P values between survey response groups. Ethics approval was received by the University of Western Ontario, Health Sciences Research Ethics Board, Research Ethics Board number 17036E).

## Results

**Figure 1:**
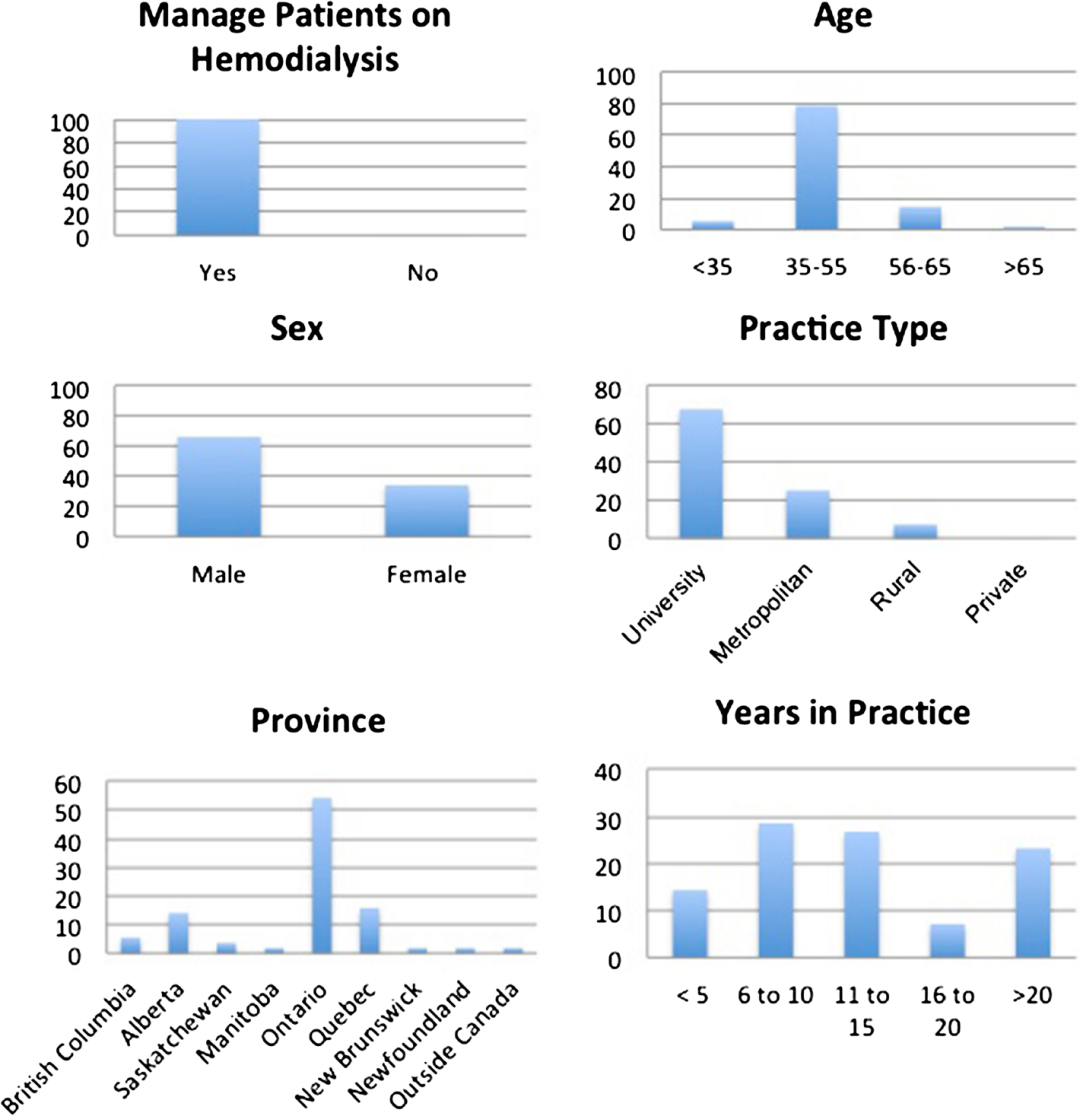
Respondent demographic factors.

A random selection of every third nephrologist member of the Canadian Society of Nephrology was sampled (n = 90), and 56 responded (62%), all of whom were responsible for the clinical care of patients on hemodialysis (Figure 1). The majority of respondents practiced in Ontario (54.4%) or Quebec (15.8%), in keeping with the large population bases of these two provinces, where 62.0% of Canada’s population resides [23]. Most had more than 11 years experience. Most respondents worked in university affiliated practices (67.8%), the remainder in the community, and none in private practice, reflecting the Canadian publicly funded health care system.

Responses to each of 6 cases are shown (Table 1). When a patient was placed on hemodialysis, uncertainty to continue warfarin to treat atrial fibrillation increased (from 16 to 36%, P = 0.0300), and the likelihood of starting warfarin decreased (from 80 to 50%, P = 0.0013). Likelihood of warfarin use increased as CHADS2 and CHA2DS2VASc score increased from 2 to 5 (from 50 to 77%, P = 0.0057), so long at bleeding risks were absent, and though explicit uncertainty was reduced at the higher scores (36% falling to 20%, P = 0.0902), significant uncertainty persisted. Half the respondents were uncertain when evaluating a scenario in which the patient was at risk for falls, (Case 4, 48%), even when there was a high stroke risk (CHADS2 = 5, CHA2DS2VASc = 8). Nephrologists were more likely to continue warfarin if there was a history of gastrointestinal bleeding, as opposed to a risk for falls (23% compared with 48%, P = 0.0099), although there was explicit uncertainty (48%, and 43%, respectively). Very few Nephrologists were likely to prescribe warfarin when a high stroke risk (CHADS2 = 5, CHA2DS2VASc = 8) was found with a history of gastrointestinal bleed and risk for falls (3.6%), while most were unlikely to continue warfarin (68%); these scenarios highlighted the difference between new prescription and discontinuation of an established therapy (3.6% prescribing if not taking v 32% continuing if already taking, P = 0.0001). Even in this high-risk scenario, with substantial agreement among nephrologists not to give a new warfarin prescription, explicit uncertainty about the decision was reported by 29% of nephrologists.

**Table 1:** Nephrologist responses, depending on stroke and fall risk, and history of GI bleed

**Case**	**CHADS2**	**CHADS-Vasc**	**Hemodialysis**	**GI bleed**	**Risk for falls**	**Likely to start warfarin (%)**	**Unlikely to start warfarin (%)**	**Uncertain (%)**
1	2	3	No	No	No	80.4	3.6	16.1
2	2	3	Yes	No	No	50.0	14.3	35.7
3	5	6	Yes	No	No	76.7	3.6	19.6
4	5	8	Yes	No	Yes	23.2	28.6	48.2
5	5	8	Yes	Yes	No	48.2	8.9	42.9
6	5	8	Yes	Yes	Yes	3.6	67.9	28.6

**Figure 2:**
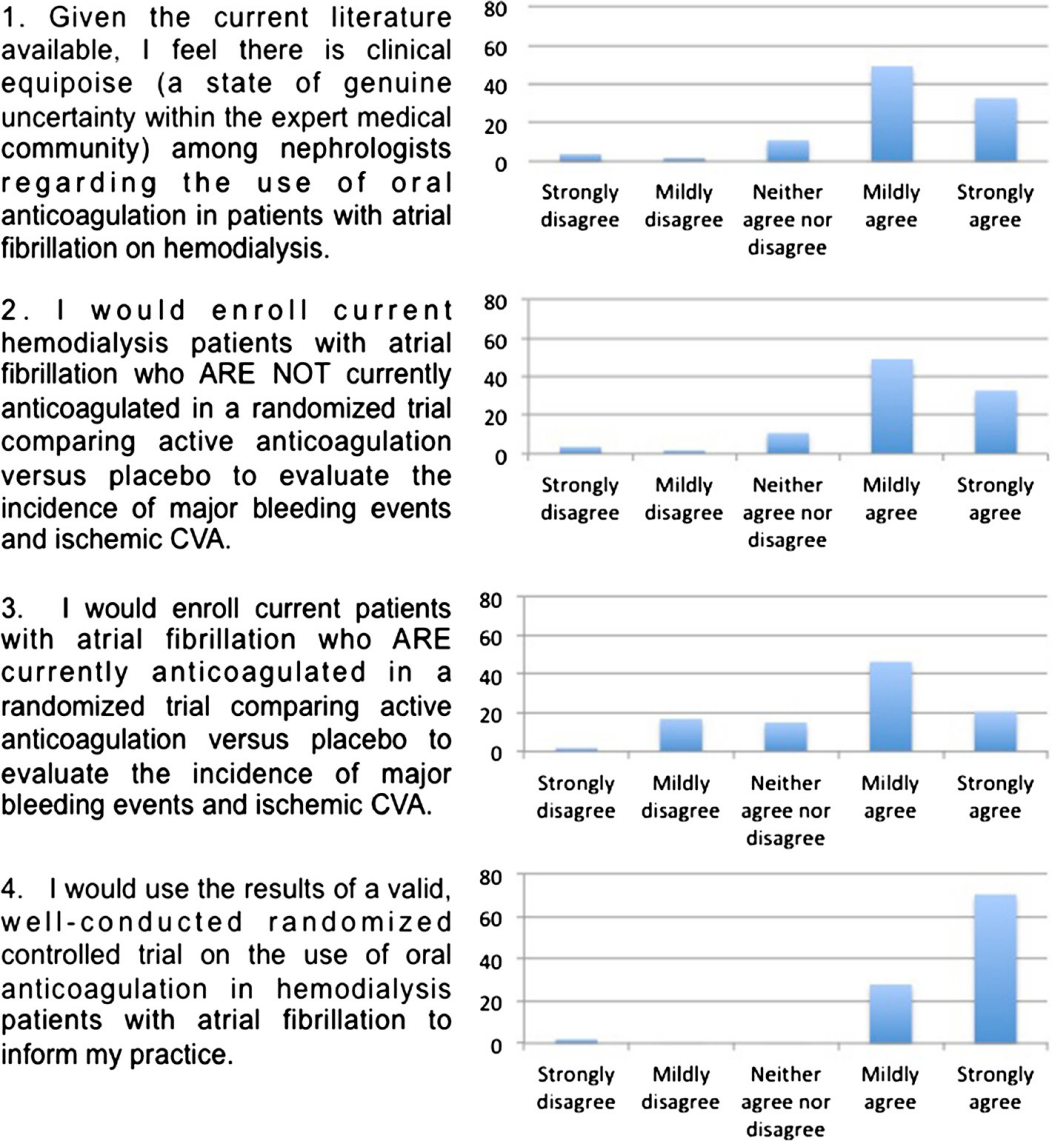
Nephrologist responses to clinical equipoise and willingness to enroll patients in randomized controlled trial.

Respondents were asked four questions at the completion of the initial survey about their feeling of clinical equipoise, willingness to enter their own patients into a randomized controlled trial and their feeling that an RCT would be useful in hemodialysis patients with atrial fibrillation (Figure 2). The majority (72%) mildly or strongly agreed that there was a state of genuine uncertainty within the expert medical community regarding the use of oral anticoagulation in hemodialysis patients with atrial fibrillation. The majority of responders mildly or strongly agreed that they would enroll patients on hemodialysis with atrial fibrillation into a randomized control trial, whether the patient is (67%) or is not (82%) currently anticoagulated. Finally, the overwhelming majority (98%) would mildly or strongly agree that the results of a valid and well-conducted RCT on the use of oral anticoagulation in hemodialysis patients with atrial fibrillation would inform their practice.

## Discussion

Our national survey of the standardized cases revealed that using warfarin in a patient on hemodialysis versus a patient with normal renal function increased Nephrologist uncertainty (Figure 1). CHADS2 and CHA2DS2VASc variables unanimously increased the likelihood of prescribing warfarin. However, in the second scenario where the patient had a very high risk for stroke and increased bleeding risks, there was much more variability and Nephrologist uncertainty. Previous gastrointestinal bleed was perceived as lower risk than a patient at risk for falls. As with real patients, Nephrologist ambiguity for warfarin use increased in patients with several comorbidities. There was not always a rationale for physician responses and the responses showed variable physician practices.

We found that asking physicians about their uncertainty directly was most revealing. An overwhelming majority (98.2%) of Nephrologists agreed that a randomized control trial in this population would inform their practice (Figure 2). Similarly, a majority of Nephrologists would enroll their own patients in such a study. Confirming our hypothesis, Nephrologists agreed (72.2%) that clinical equipoise exists among their community and that a randomized control trial on this topic is needed.

Risk thresholds are a critical component in physician decision-making [24] For example, a decision to anticoagulate a patient with atrial fibrillation must weigh the perceived risks of bleeding and stroke. However, physicians are historically inaccurate in predicting the risks in individual patients in a number of clinical contexts, even when the probabilities of specific outcomes are well defined [25-28]. Furthermore, an accurate assessment of risk requires reliable data on outcomes in the patient group of interest. The establishment of bleeding and stroke outcomes has been challenging and elusive in hemodialysis patients with atrial fibrillation. Certainly this uncertainty can be addressed only with more definitive data on bleeding, stroke and survival, by the conduction of well-designed prospective trials.

The anticoagulation of hemodialysis patients with atrial fibrillation may have unacceptable risks of bleeding [2]. On the contrary, a high proportion of hemodialysis patients have atrial fibrillation, [3,4] and the risk of CVA may or may not be decreased with warfarin use [5,6]. Certainly there would be a substantial health cost savings due to elimination of resources dedicated to monitoring, dosing and prescription of warfarin anticoagulation. However, neither KDOQI nor KDIGO offer recommendations related to chronic anticoagulation of hemodialysis patients. Equipoise reflected in this current study suggests that prospective trials of the two basic strategies (anticoagulation free versus warfarin anticoagulation), would infer the nephrology literature with better clinical practice guidelines.

There would be clear challenges in funding, designing and carrying out a randomized controlled trial on warfarin use in hemodialysis. Since warfarin is inexpensive, readily available and bioequivalent [29], the funding for such a trial would need to come from public, rather than pharmaceutical company funding. Alternatively, a newer non-Vitamin K dependent anticoagulant could be evaluated against warfarin. However, such a study would be challenging in the absence of a placebo patient group, since the question of whether any anticoagulation of any kind is required in this setting would remain unanswered [30]. Furthermore, there is limited experience and safety of newer anticoagulants in hemodialysis patients [31]. Thus, a country with sufficient public funds would need to initiate this rigorously designed, randomized controlled trial.

Designing a randomized controlled trial in this clinical setting would need to overcome ethical challenges. Specifically, randomizing patients to the use of warfarin in hemodialysis patients may be unethical if clinicians believe that treatment to be inferior to no anticoagulation. Patients may have strong preferences that limit recruitment and bias outcomes [32]. However, this study confirms that uncertainty is common in Canadian nephrologists, and that willingness to enter patients into a randomized trial is high. Thus, it would not be unethical for Canadian nephrologists to enter their patients into such a trial, since they don’t feel that anticoagulation of hemodialysis patients with atrial fibrillation is either inferior or superior. Ultimately, randomized controlled trials are the most rigorous way of determining whether a cause-effect relation exists between treatment and outcome [33], and even despite the above challenges, remains the ideal way to assess the effect of anticoagulation of hemodialysis patients with atrial fibrillation.

There are a number of limitations in our study. Firstly, the survey to the Canadian Society of Nephrologists was limited in that this survey was a novel tool that has not yet been validated. The response rate was limited, however the results still likely reflect the Canadian population of Nephrologists as the demographics of the respondents were fairly representative of our Nephrology population base (Figure 1). Secondly, newer novel oral anticoagulants were not considered in our survey. However, while data on warfarin use in hemodialysis patients is limited, data on novel oral anticoagulant use is nonexistent; thus, warfarin is likely to remain the standard of care in this clinical setting for the foreseeable future. Finally, the generalizability of these results is limited as only Canadians were surveyed. Response bias must also be accounted for when interpreting the results of the survey.

## Conclusions

The results of this study have confirmed that there is limited evidence in the literature to guide decisions on oral anticoagulation in hemodialysis patients with atrial fibrillation. This is reflected in the inconsistent practice among Nephrologists nationally. The paucity of evidence in the literature, and the uncertainty in national practice, lend credence to the performing of a randomized control trial. Indeed, Canadian Nephrologists agree there is clinical equipoise, and are willing to enroll their patients in a well designed randomized control trial.

## Abbreviations

CHADS2: Congestive heart failure (1) Hypertension (1), Age > 75 (1), Diabetes mellitus (1), Past Stroke or transient ischemic attack (2); CHA2DS2VASc: Congestive Heart Failure (1) Hypertension (1), Age > 75 (2), Diabetes Mellitus (1), Prior Stroke or TIA or thromboembolism (2), Vascular disease (Past myocardial infarction, peripheral artery disease or aortic plaque) (1), Age > 65 (1), Sex category (female gender = 1); CKD: Chronic kidney disease; CSN: Canadian society of nephrology; CVA: Cerebrovascular accident; RCT: Randomized controlled trial.

## Competing interests

There are no financial competing interests and no non-financial competing interests.

## Authors’ contributions

SJ created and distributed the survey, compiled and analyzed the results, then drafted the manuscript. BT analyzed the results, performed statistical analysis, and helped to draft and to modify the manuscript. CEL, CML, PGB participated in the conception and design of the study. LM conceived and designed the study, and helped to draft the manuscript. All authors read and approved the final manuscript.

## Pre-publication history

The pre-publication history for this paper can be accessed here:

http://www.biomedcentral.com/1471-2369/14/174/prepub
